# A Rare Case of Cerebral Amyloidoma Mimicking Thalamic Glioma in a Rheumatoid Arthritis Patient

**DOI:** 10.3390/pathophysiology32030031

**Published:** 2025-07-01

**Authors:** Elyaa Saleh, Nour Abdelaziz, Malaak Ramahi, Antonia Loukousia, Theodossios Birbilis, Dimitrios Kanakis

**Affiliations:** 1 Laboratory of Pathology, Department of Basic and Clinical Sciences, University of Nicosia Medical School, Nicosia 2408, Cyprus; saleh.ely@live.unic.ac.cy (E.S.); abdelaziz.n1@live.unic.ac.cy (N.A.); ramahi.m@live.unic.ac.cy (M.R.); 2 Laboratory of Pathology, AHEPA General Hospital, Aristotle University of Thessaloniki, 54636 Thessaloniki, Greece; tonialoukousia@gmail.com; 3 Department of Neurosurgery, Democritus University of Thrace, 67100 Alexandroupolis, Greece; birbilis@med.duth.gr; 4 Centre of Neuroscience and Integrative Brain Research (CENIBRE), University of Nicosia Medical School, Nicosia 1010, Cyprus

**Keywords:** cerebral amyloidoma, glioma, rheumatoid arthritis, systemic AA amyloidosis

## Abstract

Amyloidosis, often referred to as “the great imitator”, is a condition characterized by the abnormal deposition of amyloid proteins in various tissues, potentially leading to organ dysfunction. When these deposits localize in the brain, they can disrupt neurological function and present with diverse clinical manifestations, making diagnosis particularly challenging. Cerebral amyloidosis is a rare entity that frequently mimics other neurological disorders, often resulting in significant delays in recognition and management. This case highlights the diagnostic challenge posed by cerebral amyloidosis and underscores its unique presentation. We present the case of a 76-year-old male with a history of rheumatoid arthritis (RA) who developed progressive right-sided weakness over several months. Three years prior, he experienced numbness on the right side of his face and upper limb. Initial imaging identified a small lesion in the left thalamic region, which was originally diagnosed as a glioma. However, due to the worsening of his clinical symptoms, further evaluation was warranted. Subsequent imaging revealed lesion growth, prompting a biopsy that ultimately confirmed the diagnosis of intracerebral amyloidoma. This case underscores the necessity of considering amyloidosis in the differential diagnosis of atypical neurological deficits, particularly in patients with systemic inflammatory conditions such as RA. The initial presentation of hemiparesis resembling a stroke, coupled with non-specific imaging findings and a prior misdiagnosis of glioma, highlights the complexity of cerebral amyloidosis. Only through brain biopsy was the definitive diagnosis established, emphasizing the need for improved diagnostic modalities to facilitate early detection. Further subtyping of amyloidosis, however, requires mass spectrometry-based proteomics or immunohistochemistry to accurately identify the specific amyloid protein involved. Clinicians should maintain a high index of suspicion for cerebral amyloidosis in patients with RA who present with progressive neurological deficits and atypical brain lesions. Early recognition and accurate diagnosis are essential to guiding appropriate management and improving patient outcomes.

## 1. Introduction

Amyloidosis comprises a diverse group of disorders marked by the misfolding and accumulation of insoluble amyloid fibrils in different organs and tissues [1]. Depending on the type of precursor protein involved, amyloidosis can be classified into different subtypes, including AL amyloidosis (light-chain amyloidosis) and AA amyloidosis (secondary amyloidosis) [2]. Among these, AA amyloidosis is associated with chronic inflammatory conditions such as rheumatoid arthritis (RA), where persistent systemic inflammation leads to the overproduction of serum amyloid A (SAA) protein, which subsequently deposits as amyloid fibrils [3]. While systemic amyloidosis primarily affects the kidneys, liver, and spleen, involvement of the central nervous system (CNS) is rare [4,5].

Cerebral amyloidosis, also known as cerebral amyloidoma, is an uncommon, localized form of amyloid deposition in the brain parenchyma [4]. Unlike cerebral amyloid angiopathy (CAA), which affects cerebral blood vessels [6], cerebral amyloidoma presents as mass-like lesions that can mimic neoplastic conditions such as gliomas [7,8]. The imaging features of cerebral amyloidoma often overlap with those of intracranial tumors, making misdiagnosis a common challenge [8].

Despite advancements in neuroimaging, the diagnosis of cerebral amyloidoma remains challenging, as MRI findings can resemble gliomas, primary CNS lymphomas, or metastatic tumors [9]. While CT and MRI imaging can aid in detection, histopathological confirmation remains the gold standard for diagnosis [10]. However, due to the lack of awareness and limited reports on cerebral amyloidoma in RA patients, many cases remain undiagnosed or misclassified, highlighting a critical gap in the literature.

This case report aims to address this gap in knowledge by presenting a patient with RA-associated cerebral amyloidoma, emphasizing the diagnostic challenges, the importance of histopathological confirmation, and the clinical implications in the differential diagnosis in patients with chronic inflammatory diseases. Increasing awareness of cerebral amyloidoma as a rare neurological complication of RA may improve early recognition and prevent misdiagnosis, ultimately leading to better patient outcomes.

## 2. Case Report

A 76-year-old male patient presented to our Neurosurgical Clinic complaining of continuously aggravating symptoms of right-sided hemiparesis during the past six months.

Three years prior, he began experiencing numbness in the right side of his face and his right upper limb. The performed MRI of the brain at that time revealed the presence of a small, localized lesion left paraventricular at the thalamic region. The subsequent MRI spectroscopy considered glioma as the most possible diagnosis for the identified lesion. In this context, treatment was initiated with Levetiracetam (Keppra; 1000 mg × 2).

It should be mentioned here that, in the patient’s medical history, there was also rheumatoid arthritis (under medical treatment with methylprednisolone (Medrol; 1 × 2 per os) as well as arterial hypertension. However, there was no previous lumbar puncture for CSF evaluation, and he was never admitted to the hospital for a thorough neurological examination.

Due to his current symptomatology, the latter primarily right-sided hemiparesis, we decided to perform an MRI of the brain as an initial step. This showed an increase in the size of the above-described lesion, which was now extending up to the left pyramidal tract. A frameless stereotactic biopsy was carried out, and the samples were sent to the Pathology Department for evaluation and diagnosis.

**Figure 1:**
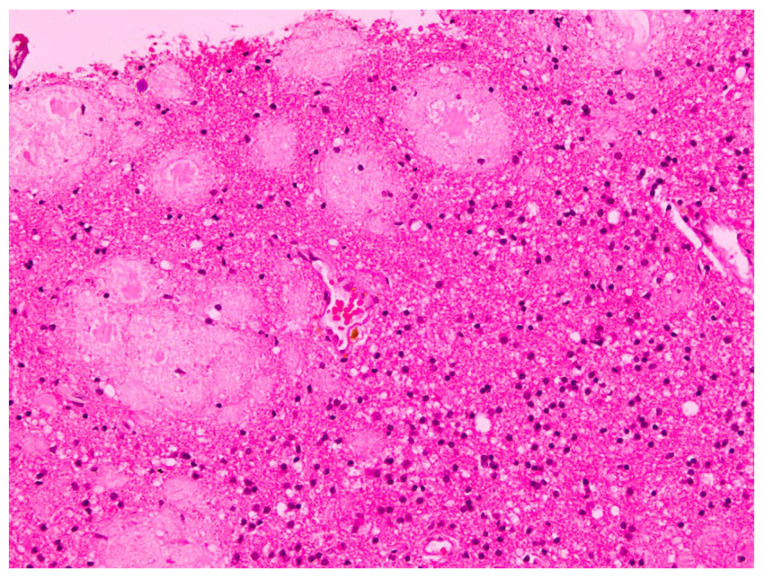
Intracerebral accumulation of amyloid, presenting as an eosinophilic amorphous material, mostly in globular shape formations of different sizes (H&E staining; magnification: ×20).

**Figure 2:**
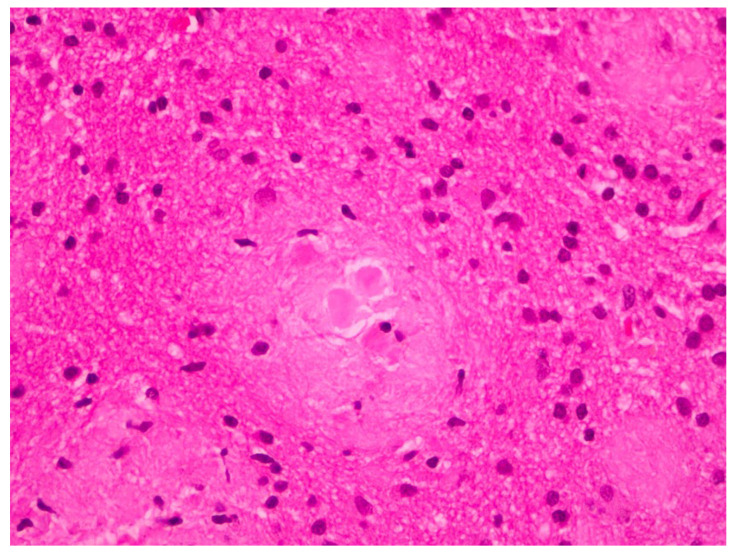
Amyloid deposits demonstrating central more dense areas (in the form of spherical cores) and less enhanced peripheral zones (H&E staining; magnification: ×40).

**Figure 3:**
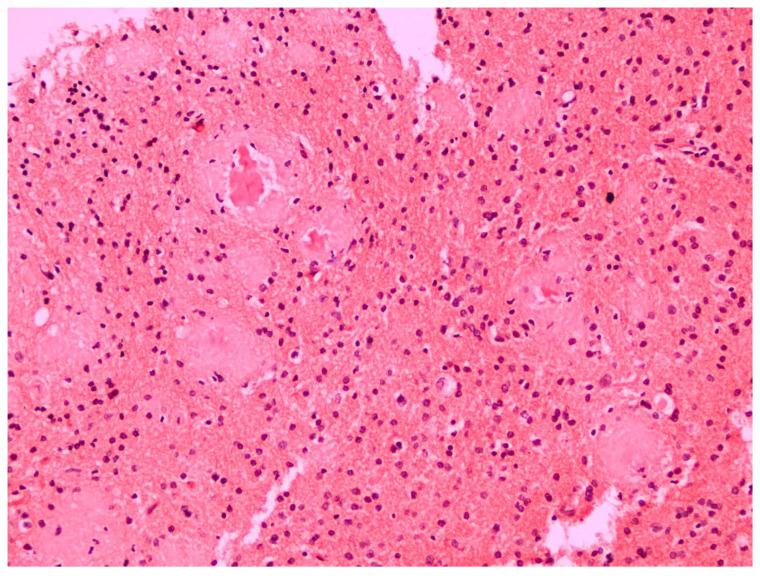
The amyloid deposits were also clearly visible in the Congo red stain (Congo red staining; magnification: ×20).

**Figure 4:**
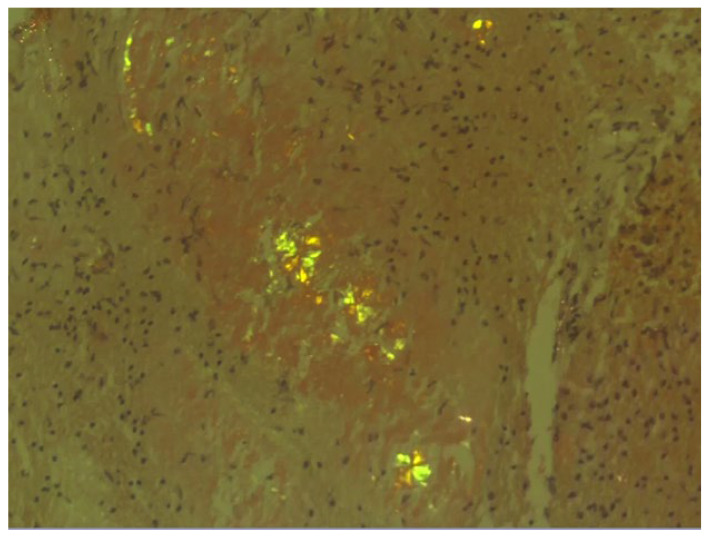
The amyloid deposits exhibited an apple-green birefringent appearance under polarized light (Congo red staining; magnification: ×20).

The histopathological examination of the specimens in the performed H&E stains showed multifocal aggregates of variable size, mostly spherical in shape, which were composed exclusively of a dense amorphous eosinophilic material. These accumulations were scattered within the resected brain parenchyma, mostly in globular shape formations (Figure 1), with more compact central areas and less enhanced peripheral zones (Figure 2). The deposits were also clearly seen in the samples subjected to Congo red staining (Figure 3), where they demonstrated an apple-green birefringent appearance under polarized light (Figure 4).

## 3. Discussion

This case highlights the difficulty in diagnosing an intracerebral amyloidoma, which initially appeared to be a glioma, a more common neurological pathology. AA amyloidosis commonly affects peripheral organs, such as the heart, kidneys, and intestine. In regions with a chronic inflammatory response of various etiologies, the incidence of AA amyloidosis is about 1–2 cases per million person-years, while the prevalence of AA amyloidosis among patients with RA ranges from 5% to 78% [11]. Previous studies have reported that 5–20% of RA patients develop AA amyloidosis, emphasizing the role of systemic inflammation in amyloid deposition, including the brain [12,13]. As far as amyloid deposits in the brain are concerned, these can be found in the leptomeninges and cortices in patients with systemic amyloidosis, including AA amyloidosis [13]. There are also reports in the literature that describe amyloid accumulation in other brain regions, such as the choroid plexus and areas with a more permeable blood–brain barrier, like the infundibulum and the area postrema [14]. A prior study analyzing 17 cases of systemic AA, AL, and Aκ amyloidosis demonstrated amyloid accumulation in these vulnerable areas, resembling deposits found in peripheral organs [14]. Additionally, nine cases of systemic AA and AL amyloidosis showed amyloid deposition in the choroid plexus [13].

**Figure 5:**
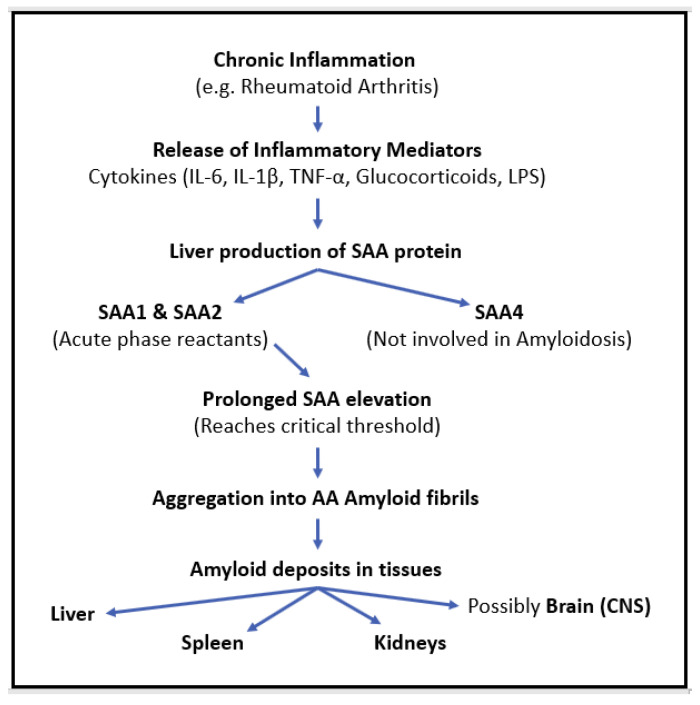
The pathophysiological cascade of AA amyloidosis in chronic inflammation.

Long-standing inflammation, as observed in RA, induces the release of proinflammatory mediators such as glucocorticoids; lipopolysaccharides (LPSs); and cytokines, including interleukin-6 (IL-6), interleukin-1β (IL1β), and tumor necrosis factor-α (TNF-α) [15]. Persistent activation of these inflammatory cytokines leads to the production of serum amyloid A (SAA) protein, an acute-phase reactant synthesized in the liver. SAA is a protein that can misfold and increase up to 1000-fold during inflammation [16]. The prolonged elevation of SAA levels can eventually reach a critical threshold over time, which becomes prone to aggregation to insoluble AA amyloid fibrils, contributing to amyloid deposition in tissues [16,17]. This pathological process contributes to systemic amyloid deposition in the kidneys, liver, and spleen, which are the main organs affected in RA-associated amyloidosis [18] (Figure 5).

The primary acute-phase isoforms of SAA (A-SAA) include SAA1 and SAA2, which are induced by proinflammatory cytokines and serve as precursors for AA amyloid fibril formation, whereas SAA4 is constitutively expressed and not directly linked to inflammation-driven amyloid deposition [19]. A-SAA plays a significant role in inflammatory diseases. Compared to C-reactive protein (CRP), A-SAA serves as a more sensitive and specific marker of inflammation in RA [20]. Studies have demonstrated that individuals with RA exhibit elevated levels of A-SAA mRNA and protein in synovial fluid compared to healthy controls [21]. A-SAA found in synovial fluid is attributed to local inflammation, which sets off the inflammatory cascade and promotes angiogenesis and Pannus formation (abnormal tissue growth in the joints) [22]. Prolonged inflammation leads to the ongoing release of proinflammatory cytokines, exacerbating disease progression. Although the short-term release of A-SAA facilitates tissue repair, its prolonged, long-term release drives chronic inflammation, which results in tissue degradation and joint erosion in RA [19]. Beyond the joints, recent research suggests that SAA deposition and persistent inflammation may extend to the central nervous system. Cognitive decline and neurodegenerative changes in RA patients have been linked to neuroinflammation, blood–brain barrier (BBB) dysfunction, and possibly SAA-mediated amyloid deposition [23].

Radiologically, cerebral amyloidoma presents as a well-defined lesion with variable imaging characteristics. On T1-weighted MRI, lesions may appear isointense or hypointense, while on T2-weighted sequences, they can be hyperintense or hypointense. Contrast enhancement patterns are also variable, ranging from minimal to peripheral enhancement. These diverse imaging features can make it challenging to differentiate cerebral amyloidoma from other intracranial pathologies, such as neoplasms or inflammatory processes [24]. Advanced MRI techniques, including susceptibility-weighted imaging (SWI) and diffusion-weighted imaging (DWI), can provide additional insights. SWI may reveal microhemorrhages due to associated vascular involvement, while DWI typically shows no restriction, helping to distinguish cerebral amyloidoma from high-grade neoplasms or ischemic lesions [25]. The absence of significant perilesional edema and mass effect further differentiates cerebral amyloidoma from more aggressive intracranial pathologies. However, due to its variable imaging characteristics, histopathological confirmation remains essential for an accurate diagnosis.

Histopathological examination remains the gold standard for confirming the diagnosis of cerebral amyloidoma [26]. Congo red staining is essential for identifying amyloid deposits, as it demonstrates the characteristic apple-green birefringence under polarized light, which serves as a definitive diagnostic marker [1]. Although precise amyloid subtyping by mass spectrometry or immunohistochemistry using amyloid-specific antibodies is currently regarded as the most reliable technique for amyloid classification [10,27], such analyses could not be performed in our case due to the unavailability of remaining tissue following the patient’s death. Despite this limitation, the diagnosis of AA amyloidosis remains the most plausible, based on the presence of severe, long-standing rheumatoid arthritis, which is a condition strongly associated with systemic AA amyloid deposition [12]. Moreover, there was no clinical, radiological, or histopathological evidence suggestive of alternative amyloid types, such as AL, Aβ, or ATTR amyloidosis. While we acknowledge the absence of confirmatory proteomic analysis, the convergence of the clinical context and histopathological findings supports our conclusion that the cerebral amyloidoma in this case most likely represents a manifestation of AA amyloidosis.

While amyloid PET scans can detect amyloid deposits, they are typically reserved for Alzheimer’s-related beta-amyloid plaques, limiting their broader application in diagnosing other forms of amyloidosis [28]. Catafau & Bullich (2015) highlight the specificity of amyloid PET imaging in detecting amyloid pathology in vivo, particularly through radiotracers such as florbetaben, florbetapir, and Pittsburgh compound B (PiB) [28]. Although primarily utilized for Alzheimer’s-related amyloidosis, emerging evidence suggests its potential role in differentiating cerebral amyloidoma from other mass-like lesions, such as gliomas or metastases [28]. In this case, the absence of amyloid PET imaging limited insight into the pattern of amyloid deposition. PET imaging with specially developed tracers, or “amyloid PET imaging”, must improve discrimination between cerebral amyloidoma and neoplastic lesions. Non-invasive diagnostic accuracy may be improved in future studies that include PET imaging, along with reduced dependence on histopathological investigations [28].

Cerebral amyloidoma may arise from various systemic amyloidosis subtypes, including AL amyloidosis, AA amyloidosis, or even localized amyloid deposits in the brain without systemic involvement. Expanding the use of amyloid PET scans could facilitate early detection and characterization of these deposits [14]. Notably, PET imaging may offer a non-invasive means of evaluating the extent of cerebral amyloid deposition, aiding in both initial diagnosis and longitudinal disease monitoring. This could be particularly valuable in cases where surgical biopsy poses a high risk or is not feasible.

The mass-like radiographic appearance of cerebral amyloidoma often leads to misdiagnosis as a glioma due to their resemblance. In our patient, the lesion’s extension along the pyramidal tract initially mimicked a tumor, leading to a misdiagnosis of a glioma. While PET imaging may help differentiate cerebral amyloidoma from neoplastic lesions through distinct tracer uptake patterns [28], it should not replace histopathological confirmation. Unlike Alzheimer’s patients, individuals with cerebral amyloidoma may lack cognitive impairment, making biopsy essential for accurate diagnosis. Given its overlap with gliomas, CNS lymphomas, and metastatic tumors, histopathological evaluation remains the gold standard. In this case, biopsy allowed for direct visualization of the characteristic birefringence of amyloid deposits, ruling out alternative pathologies [29]. Despite imaging advancements, histopathological analysis following surgical intervention remains indispensable for definitive diagnosis [26].

The management of AA amyloidosis in rheumatoid arthritis (RA) primarily focuses on controlling the underlying inflammatory process, thereby reducing the production of SAA and limiting amyloid deposition. The mainstay of treatment involves disease-modifying anti-rheumatic drugs (DMARDs), including methotrexate, sulfasalazine, and leflunomide, which help suppress chronic inflammation [30]. In addition to conventional DMARDs, biological therapies play a crucial role in modulating the inflammatory pathways involved in AA amyloidosis. Tumor necrosis factor (TNF) inhibitors, such as etanercept and adalimumab, and interleukin-6 (IL-6) inhibitors, such as tocilizumab, are widely used in RA management. Notably, IL-6 inhibitors have demonstrated greater efficacy than TNF inhibitors in managing AA amyloidosis, as IL-6 is directly involved in stimulating SAA production [31].

Corticosteroids, such as methylprednisolone (Medrol), are commonly used for symptomatic relief and inflammation control but are not the preferred long-term option due to their limited effect on SAA production and potential complications such as osteoporosis and metabolic disturbances [32]. In this case, the patient was receiving levetiracetam (Keppra, 1000 mg × 2) for seizure control and methylprednisolone (1 × 2 per os) for RA management but was not on TNF or IL-6 inhibitors, which may have contributed to continued amyloid deposition and disease progression.

Given the mass-like nature of cerebral amyloidosis, surgical resection is one of the primary treatment options in symptomatic cases, as it can relieve neurological deficits due to mass effect [26]. While complete resection is often curative, some cases of recurrence have been documented, necessitating long-term follow-up [33]. In instances where the lesion is inoperable due to its location, radiotherapy has been explored as an alternative to halt progression [34]. However, due to the rarity of cerebral amyloidoma, there is no standardized treatment protocol, and management must be individualized based on lesion location, symptom severity, and patient comorbidities.

We fully recognize the diagnostic limitations of this case and acknowledge that the absence of molecular confirmation necessitates a cautious interpretation of the presumed amyloid subtype. A thorough differential diagnosis must therefore be considered. While the clinical context strongly favors AA amyloidosis—given the patient’s history of long-standing, severe rheumatoid arthritis—other amyloid types cannot be definitively excluded: *AL (light chain) amyloidosis* is a common systemic form; however, there was no evidence of plasma cell dyscrasia or monoclonal gammopathy, and no laboratory or systemic findings suggestive of AL were documented. *Aβ (beta-amyloid) deposition*, typically seen in Alzheimer’s disease or cerebral amyloid angiopathy (CAA), usually affects elderly individuals with cognitive decline and involves the cortical vasculature or parenchyma. In our case, the patient exhibited no cognitive symptoms, and the lesion was localized to the deep brain (thalamus), which is atypical for Aβ-related pathology. Moreover, histological examination revealed no neurofibrillary tangles or neuritic plaques. *ATTR (transthyretin) amyloidosis*, whether hereditary or wild-type, most often presents with cardiac involvement or peripheral neuropathy in older adults—features not observed in our patient. *Hereditary amyloidosis* cases, such as those associated with mutations in gelsolin, cystatin C, or lysozyme, typically occur in younger individuals or as part of familial syndromes. No suggestive clinical or family history was identified in this case.

Histologically, the presence of Congo red stained deposits, which exhibited an apple-green birefringent appearance under polarized light, confirmed amyloid. The lesion’s radiological and pathological features were compatible with cerebral amyloidoma, a rare, typically localized form of amyloidosis. In the absence of definitive subtyping, the diagnosis of AA-type cerebral amyloidoma remains presumptive but is supported by the inflammatory background, exclusion of other types, and lack of systemic findings.

In summary, while we acknowledge the diagnostic limitations and the inability to perform confirmatory molecular subtyping, the convergence of clinical, radiological, and histopathological findings points toward a likely diagnosis of cerebral amyloidoma in the setting of presumed AA amyloidosis. Nevertheless, alternative amyloid types must be considered in similar cases, and definitive tissue typing should be pursued whenever feasible to guide diagnosis and management.

## 4. Conclusions

Although uncommon, cerebral amyloidoma is a possible manifestation in patients with RA and can easily be overlooked in favor of more common neurological disorders, such as neoplastic conditions like gliomas. This underreporting highlights the importance of clinicians considering cerebral amyloidoma in the differential diagnosis of brain lesions, particularly in RA patients with atypical imaging or clinical features. A comprehensive diagnostic work-up, including advanced imaging and, when necessary, biopsy, is essential to prevent misdiagnosis and ensure accurate management. Further research is needed to better understand the link between RA, systemic AA amyloidosis, and cerebral involvement, as well as to explore the potential role of advanced imaging and biomarkers in improving early detection, diagnostic accuracy, and therapeutic decision-making for this rare but clinically significant condition.

## Data Availability

No new data were created or analyzed in this study. Data sharing is not applicable to this article.

## Abbreviations

The following abbreviations are used in this manuscript:

**Abbreviations**	**Full forms**
RA	Rheumatoid arthritis
CA	Cerebral amyloidosis
SAA	Serum amyloid alpha
CSF	Cerebrospinal fluid
H&E	Hematoxylin and eosin
LPSs	Lipopolysaccharides
IL	Interleukin
TNF	Tumor necrosis factor
CAA	Cerebral amyloid angiopathy
DMRADs	Disease-modifying anti-rheumatic drugs
